# The Compensation Index Is Better Associated with DSA ASITN Collateral Score Compared to the Cerebral Blood Volume Index and Hypoperfusion Intensity Ratio

**DOI:** 10.3390/jcm12237365

**Published:** 2023-11-28

**Authors:** Dhairya A. Lakhani, Aneri B. Balar, Manisha Koneru, Sijin Wen, Meisam Hoseinyazdi, Cynthia Greene, Risheng Xu, Licia Luna, Justin Caplan, Adam A. Dmytriw, Adrien Guenego, Max Wintermark, Fernando Gonzalez, Victor Urrutia, Judy Huang, Kambiz Nael, Ansaar T. Rai, Gregory W. Albers, Jeremy J. Heit, Vivek S. Yedavalli

**Affiliations:** 1 Department of Radiology and Radiological Sciences, Johns Hopkins University, Baltimore, MD 21218, USAmhosein3@jhmi.edu (M.H.); vyedava1@jhmi.edu (V.S.Y.); 2 Cooper Medical School, Rowan University, Camden, NJ 08028, USA; 3 Department of Biostatistics, West Virginia University, Morgantown, WV 26506, USA; 4 Department of Neurosurgery, Johns Hopkins University, Baltimore, MD 21218, USAjustincaplan@jhmi.edu (J.C.); fernando.gonzalez@jhu.edu (F.G.);; 5 Department of Radiology, Harvard Medical School, Boston, MA 02115, USA; 6 Department of Radiology, Université Libre De Bruxelles Hospital Erasme, 1070 Anderlecht, Belgium; 7 Department of Radiology, University of Texas, MD Anderson Center, Houston, TX 77030, USA; 8 Department of Neurology, Johns Hopkins University, Baltimore, MD 21218, USA; 9 Division of Neuroradiology, David Geffen School of Medicine, University of California Los Angeles, Los Angeles, CA 90095, USA; 10 Department of Radiology, West Virginia University, Morgantown, WV 26506, USA; 11 Department of Radiology, Stanford University School of Medicine, Stanford, CA 94063, USAjheit@stanford.edu (J.J.H.)

**Keywords:** acute ischemic stroke, compensation index, collateral status, CBV index, hypoperfusion intensity ratio, HIR

## Abstract

Background: Pretreatment CT Perfusion (CTP) parameters serve as reliable surrogates of collateral status (CS). In this study, we aim to assess the relationship between the novel compensation index (CI, Tmax > 4 s/Tmax > 6 s) and already established CTP collateral markers, namely cerebral blood volume (CBV) index and Hypoperfusion Intensity Ratio (HIR), with the reference standard American Society of Interventional and Therapeutic Neuroradiology (ASITN) collateral score (CS) on DSA. Methods: In this retrospective study, inclusion criteria were the following: (a) CT angiography confirmed anterior circulation large vessel occlusion from 9 January 2017 to 10 January 2023; (b) diagnostic CT perfusion; and (c) underwent mechanical thrombectomy with documented DSA-CS. Student *t*-test, Mann–Whitney-U-test and Chi-square test were used to assess differences. Spearman’s rank correlation and logistic regression analysis were used to assess associations. *p* ≤ 0.05 was considered significant. Results: In total, 223 patients (mean age: 67.8 ± 15.8, 56% female) met our inclusion criteria. The CI (ρ = 0.37, *p* < 0.001) and HIR (ρ = −0.29, *p* < 0.001) significantly correlated with DSA-CS. Whereas the CBV Index (ρ = 0.1, *p* > 0.05) did not correlate with DSA-CS. On multivariate logistic regression analysis taking into account age, sex, ASPECTS, tPA, premorbid mRS, NIH stroke scale, prior history of TIA, stroke, atrial fibrillation, diabetes mellitus, hyperlipidemia, heart disease and hypertension, only CI was not found to be independently associated with DSA-CS (adjusted OR = 1.387, 95% CI: 1.09–1.77, *p* < 0.01). Conclusion: CI demonstrates a stronger correlation with DSA-CS compared to both the HIR and CBV Index where it may show promise as an additional quantitative pretreatment CS biomarker.

## 1. Background

Collateral status (CS) is an excellent biomarker associated with ischemic core growth and success of reperfusion in patients with anterior circulation acute ischemic stroke caused by large vessel occlusion (AIS-LVO) [1,2,3,4,5].

Pretreatment CT perfusion (CTP) parameters of the hypoperfusion intensity ratio (HIR) and cerebral blood volume (CBV) index are validated using pretreatment imaging surrogates of CS [6,7,8,9,10,11]. The CBV index is defined as the average CBV in Tmax > 6 s region compared to the average CBV in normal brain tissue [12]. Hypoperfusion intensity ratio (HIR) was defined as the volume of tissue with Tmax > 10 s divided by the volume of tissue with Tmax > 6 s [13]. Both the CBV Index and HIR parameters have each shown a correlation with infarct growth and with clinical outcomes following mechanical thrombectomy (MT) [14,15,16,17,18,19].

Although these CTP markers of CS have been previously validated, none take into account the volume of tissue with a perfusion delay of Tmax > 4 s, which is thought to represent benign oligemia [20]. The volume of benign oligemia directly relates to autoregulation by vasodilation, which is dependent on the patient’s CS [20]. We postulate that Tmax > 4 s may also be a marker of CS where patients with higher Tmax > 4 s and lower Tmax > 6 s volumes are considered a favorable compensatory response through collateral routes. We therefore present the compensation index (CI) as the volume of tissue with Tmax > 4 s divided by the volume of tissue with Tmax > 6 s as a novel quantitative pretreatment CS biomarker.

In this study, we aimed to investigate the relationship between the CBV index, HIR and CI as pretreatment CTP markers of CS with the reference standard ASITN CS. We hypothesize that the CI correlates as well as existing markers of CTP collateral status CBV index and HIR with CS on DSA.

## 2. Methods

### 2.1. Study Design

We performed a retrospective analysis of prospectively maintained stroke databases, and we identified consecutive patients from two comprehensive stroke centers from 29 July 2019 to 10 January 2023 who met our inclusion criteria. This study was approved through the Johns Hopkins Institutional Review Board (IRB00269637) and follows the STROBE checklist guidelines as an observational study [21].

### 2.2. Study Participants

The inclusion criteria for this study were the following: (a) MT triage within 24 h of symptom onset or last known well; (b) diagnostically adequate multimodal pretreatment CT imaging including noncontrast CT (NCCT), CT angiography (CTA) and CTP; (c) AIS due to a CTA confirmed large vessel occlusion of proximal supraclinoid ICA, ICA terminus, MCA occlusion, specifically including M1 and proximal M2 segments of the MCA [22]; and (d) who underwent MT and had recorded ASITN CS [23].

This study was conducted in accordance with the Declaration of Helsinki and the Health Insurance Portability and Accountability Act (HIPAA). Informed consent was waived by the institutional review boards given the retrospective study design. The decisions to administer IV thrombolysis and/or perform MT were made on an individual basis based on consensus stroke team evaluation per institutional protocols.

### 2.3. Data Collection

Baseline and clinical data were collected through electronic records and stroke center databases for each patient including demographics, site of occlusion, laterality of occlusion, TOAST classification [24] and baseline CTP parameters at first presentation.

### 2.4. CTP Image Acquisition

Whole brain pretreatment CTP was performed on the Siemens Somatom Force (Erlangen, Germany) with the following parameters: 70 kVP, 200 effective mAs, rotation time 0.25 s, average acquisition time 60 s, collimation 48 × 1.2 mm, pitch value 0.7, 4D range 114 mm × 1.5 s. CTP images are then automatically processed using RAPID, a commercial FDA-approved AI software (iSchemaView, Menlo Park, CA, USA) for generating quantitative perfusion maps.

### 2.5. Image Analysis

All the CTPs were assessed by board-certified neuroradiologists with 9 years of working experience for diagnostic adequacy of the CTPs, where only those deemed diagnostic adequate were included in the study. CTP images were then post-processed using RAPID commercial software (IschemaView, Menlo Park, CA, USA) for generating Tmax and CBV maps, from which the CBV Index, HIR, Tmax > 4 s, and Tmax > 6 s were calculated. CI was defined as the ratio of the Tmax > 4 s volume divided by the Tmax > 6 s volume.

The compensation index (CI) was calculated as the volume of tissue with Tmax > 4 s divided by the volume of tissue with Tmax > 6 s as a novel quantitative pretreatment CS biomarker.

The ASITN CS was independently assessed by a board-certified neuroradiologist and the performing neurointerventionalist. Any discrepancies were resolved based on a consensus review. ASITN grades included the following: Grade 0, no collaterals visible to the ischemic region; Grade 1, slow collaterals to the periphery with persisting defect; Grade 2, rapid collaterals to the periphery with persisting defect; Grade 3, slow-but-complete collateral flow to the ischemic territory; and Grade 4, rapid and complete collateral flow to the ischemic territory [23].

### 2.6. Statistical Analysis

The objective of this study is to assess the association between CI, CBV Index, HIR and DSA CS. Descriptive statistics were used to summarize patient data. Categorical data was described using contingency tables including counts and percentages; continuous variables were summarized with mean (± Standard Deviation) or median (range). A student *t*-test was used in the data analysis for continuous variables, the Mann–Whitney U test was used in the data analysis for ordinal data and the Chi-square test was used for categorical data.

Spearman correlation analysis was used to assess the correlation between CI, CBV Index and HIR with CS based on DSA CS. Statistically significant analysis was described as *p* < 0.05, *p* < 0.01 and *p* < 0.001.

A logistic model was used to estimate the odds ratio between CI, CBV Index, and HIR when the ASITN CS score was dichotomized as a binary variable (0–2 versus 3–5). In the multivariate data analysis, the adjusted odds ratio between CI, CBV Index and HIR with DSA CS score was estimated using a multiple logistic regression model, adjusting for any potential confounding variables. *p* ≤ 0.05 was considered significant.

## 3. Results

A total of 223 consecutive patients (mean age: 67.77 ± 15.76, 56.1% female) met our inclusion criteria. In total, 80 patients (35.9%) received intravenous tissue plasminogen activator administered (IV tPA).

Of 223 patients, 162 (72.7%) had M1 segment occlusion, 60 (19.3%) had proximal M2 segment occlusion and 16 (7.2%) had ICA supraclinoid segment occlusion. Patient demographic and stroke treatment details are presented in Table 1.

## 4. Spearman Correlation Analyses

### 4.1. CI and ASITN Collateral Score

Distribution of CI in patients with good and poor CS defined by ASITN CS is illustrated in Table 1.

**Figure 1:**
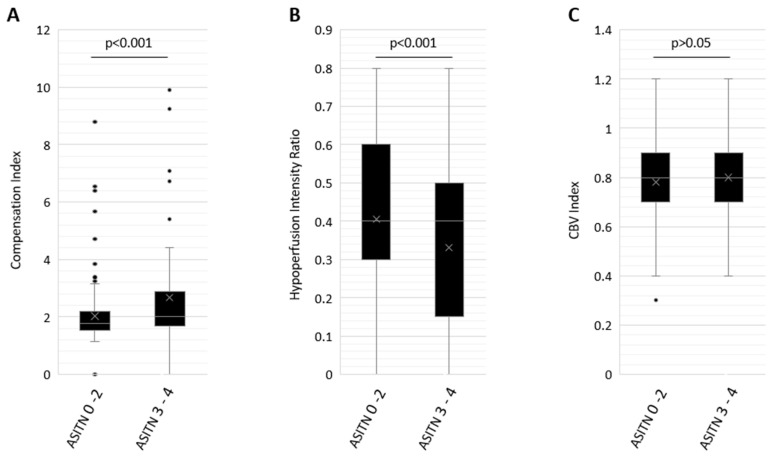
Box and whisker plot distribution of Compensation Index (CI, (**A**)), Cerebral Blood Volume Index (CBV Index, (**B**)) and Hypoperfusion Index (HIR, (**C**)) in patients with poor DSA CS of 0–2 and robust DSA CS of 3 or greater. Box plot represents median and interquartile range, whereas whiskers represent minimum and maximum values.

**Figure 2:**
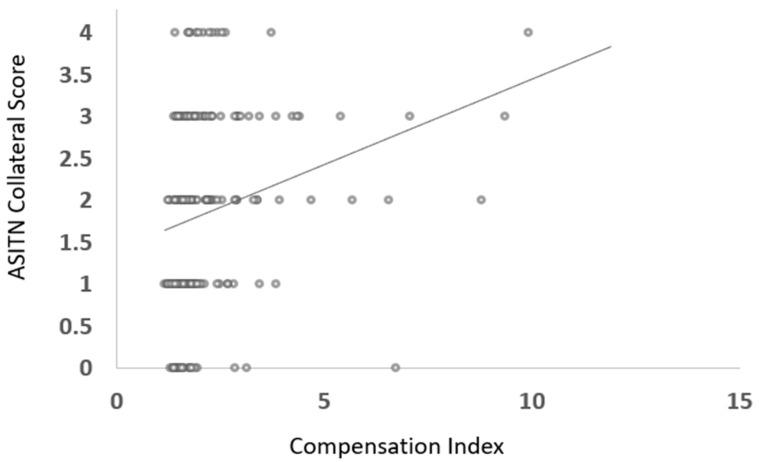
Scatter plot illustrating distribution of compensation index and ASITN collateral score in patients with anterior circulation large vessel occlusion.

Mean CI in patients with good DSA CS was 2.67 ± 1.85 and with poor DSA CS was 2.03 ± 1.04, *p* < 0.001 (Figure 1). A modest, significant positive correlation was observed between the compensation index and ASITN CS (ρ = 0.37, *p* < 0.001) (Figure 2).

Univariable logistic regression analysis to assess the association of CI with good DSA CS showed OR of 1.39 (95% CI: 1.10–1.74, *p* < 0.001). On multivariate logistic regression analysis taking into account sex, age, ASPECTS, tPA, premorbid mRS, NIH stroke scale, prior history of TIA, stroke, atrial fibrillation, diabetes mellitus, hyperlipidemia, heart disease and hypertension, CI was found to be independently associated with DSA CS (adjusted OR = 1.387, 95% CI: 1.09–1.77, *p* < 0.01 (Table 2).

### 4.2. HIR and ASITN Collateral Score

Distribution of HIR in patients with good and poor CS defined by ASITN CS is illustrated in Table 1.

**Figure 3:**
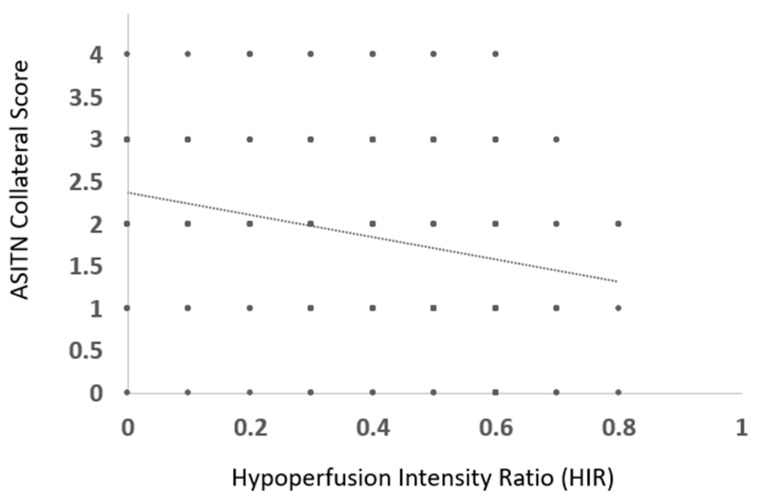
Scatter plot illustrating distribution of HIR with ASITN collateral score in patients with anterior circulation large vessel occlusion.

Mean HIR in patients with good DSA CS was 0.32 ± 0.22 and with poor DSA CS was 0.41 ± 0.21, *p* < 0.05 (Figure 1). A weak, significant negative correlation was observed between HIR and ASITN CS (ρ = −0.29, *p* < 0.001) (Figure 3).

Univariable logistic regression analysis to assess for association of HIR with good DSA CS showed OR of 3.37 (95% CI: 2.0–5.65, *p* < 0.05). On multivariate logistic regression analysis taking into account sex, age, ASPECTS, tPA, premorbid mRS, NIH stroke scale, prior history of TIA, stroke, atrial fibrillation, diabetes mellitus, hyperlipidemia, heart disease and hypertension, HIR was not found to be independently associated with DSA CS (adjusted OR = 0.38, *p* = 0.22, 95% CI: 0.08–1.76) (Table 3).

### 4.3. CBV Index and ASITN Collateral Score

The distribution of the CBV Index in patients with good and poor CS defined by ASITN CS is illustrated in Table 1.

**Figure 4:**
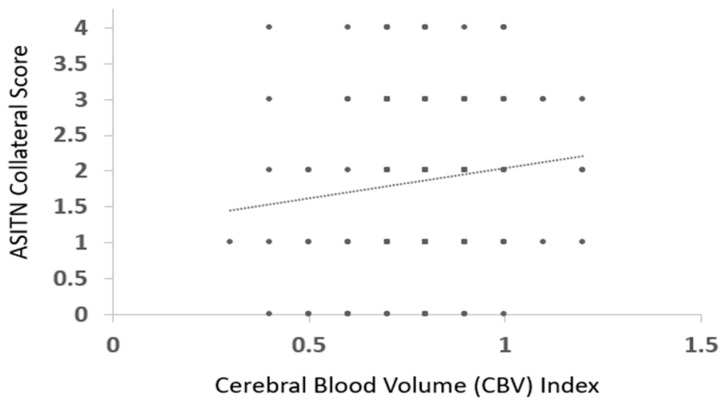
Scatter plot illustrating distribution of CBV index with ASITN collateral score in patients with anterior circulation large vessel occlusion.

Mean CBV Index in patients with good DSA CS was 0.80 ± 0.16, and with poor DSA CS was 0.78 ± 0.15, *p* > 0.05 (Figure 1). No significant correlation was observed between the CBV index and ASITN CS (ρ = 0.1, *p* > 0.05) (Figure 4).

Univariable logistic regression analysis to assess the association of the CBV Index with good DSA CS showed an OR of 2.09 (95% CI: 0.36–12.25, *p* = 0.42).

## 5. Discussion

Our study demonstrates that CI better correlates with the reference standard DSA CS than the HIR and CBV index. Furthermore, when accounting for confounders in multivariable regression analysis, only the CI was independently associated with DSA CS. This is the first study exploring associations of CI, as a baseline CTP CS parameter, with DSA CS.

Good CS is associated with higher chances of successful recanalization following MT, better penumbra salvage and favorable functional outcomes [16,25,26,27,28,29,30,31,32,33]. Hence, assessing CS before treatment can aid in prognostication for AIS-LVO patients. Subjective assessment of CTA data has a wide range of inter- and intra-user agreement [34,35,36], making it critical to use more objective markers in decision making. Prior studies have validated certain pretreatment quantitative CTP parameters as surrogates of CS and predictors of clinical outcomes [37,38,39,40,41,42,43,44,45,46,47].

Of the previously described quantitative CTP CS parameters, HIR has been the most studied. Several prior studies have demonstrated that HIR can serve as a reliable CS marker [19,48,49]. HIR correlation with ASITN CS is well established, with studies showing a statistically significant correlation. The degree of correlation has been reported over a range of modest association (coefficient (r) = −0.33) [48] to moderate association (coefficient (r = −0.76) [19,49]. Our correlation was similar to Guenego et al. [48], whereas it was lower than what was reported by Kurmann et al. [19] and Ai et al. [49]. The wide range of variation in the correlation coefficient is multifactorial. First, the patient selection is different across different studies. Kurmann et al. [19] included M1 and M2 segment occlusions, Ai et al. [49] included all patients who underwent MT and Guenego et al. [48] included only M1 occlusions, whereas, in our cohort, we report all patients with anterior circulation large vessel occlusion. Second, the sample size ranged from 30 to 115 patients; thus, ours has the largest sample size of 223.

CBV index has also shown a good correlation in predicting the infarct growth, and clinical and functional outcomes following MT in AIS-LVO patients [16,50]. However, to date, no studies have directly evaluated the relationship between the CBV index and ASITN CS. In our study, we did not find any significant correlation between the CBV index and ASITN CS. This could stem from the fact that, by definition, the CBV index takes into account venous collaterals. Whereas ASITN CS measured following intra-arterial contrast injection is heavily dependent on the arterial flow, and may not be imaged long enough through the venous phase to capture that portion of the collateral cascade, this may factor out the venous collaterals in CS estimation [26,51].

In our study, we present a novel quantitative pretreatment CTP biomarker of CS with the CI, which utilizes Tmax > 4 s volume. Tmax > 4 s volume is thought to mainly represent benign oligemia or tissue that is hypoperfused but will not go onto infarct in the absence of intervention [20]. However, we postulate that the Tmax > 4 s volume can also reflect a component of CS. We assert that CI, as a ratio of Tmax > 4 s volume divided by Tmax > 6 s volume, is a surrogate for collateral-based compensatory response where a higher ratio represents a more favorable profile. We surmise that the difference in volume representing benign oligemia to penumbra likely relates to autoregulation, driven by vasodilation from collateral routes. This vasodilatory response would require a robust collateral supply that prevents the already hypoperfused tissue from reaching critical levels [20].

There are several limitations of this study to acknowledge. First, this study is hypothesis generating with the use of a convenience sample that was retrospectively reviewed from our prospectively collected database. Second, our analysis is restricted to the use of one commercial software platform and two comprehensive stroke centers. Third, there is a modest inter- and intra-observer agreement of ASITN CS [52]. Our study is, however, strengthened by our sample size of 223 derived from our prospectively maintained databases.

Our study lays the foundation for larger, more robust studies further assessing CI as an imaging biomarker of CS. Future studies may evaluate the relationship between CI and risk of post-procedural hemorrhagic transformation and clinical outcomes. For instance, with several recent trials showing the efficacy of MT with large cores [53,54,55,56], assessment of CS in this subpopulation may provide additional value, particularly in regard to rapid infarct growth.

Future studies are needed to expand our understanding of the adjunct role of CI with other similar pretreatment CTP-based markers in clinical evaluation and decision making in patients with AIS-LVO.

## Figures and Tables

**Table 1:** Demographics of study participants.

Study Demographics	Total (*n* = 223)	DSA ASITN CS (0–2) (*n* = 151)	DSA ASITN CS (3–4) (*n* = 72)	*p* Value
Age in Years (mean ± standard deviation)	67.77 ± 15.76	68.17 ± 15.90	66.93 ± 15.53	0.583
Sex (Numbers (%))				0.636
Female	125 (56.05%)	83 (54.97%)	42 (58.33%)	
Male	98 (43.95%)	68 (45.03%)	30 (41.67%)	
Race (Numbers (%))				0.268
African American	90 (40.36%)	62 (41.06%)	28 (38.89%)	
Caucasian	117 (52.47%)	78 (51.66%)	39 (54.17%)	
Asian	7 (3.14%)	3 (1.99%)	4 (5.56%)	
Other	9 (4.04%)	8 (5.30%)	1 (1.39%)	
Pertinent Details at Presentation				
Prior history of stroke or Transient Ischemic Attack (Numbers (%))	44 (19.73%)	29 (19.21%)	15 (20.83%)	0.775
Admission NIH Stroke Scale (mean ± standard deviation)	15.65 ± 6.82	16.30 ± 6.82	14.29 ± 6.66	0.040 *
Premorbid modified Rankin score (mRS) (mean ± standard deviation)	0.63 ± 1.07	0.55 ± 1.02	0.80 ± 1.14	0.105
Admission Alberta stroke program early CT score (ASPECTS) (mean ± standard deviation)	8.64 ± 1.86	8.43 ± 2.03	9.07 ± 1.30	0.005 *
Intravenous tissue plasminogen activator administered (IV tPA) (Numbers (%))	80 (35.87%)	51 (33.77%)	29 (40.28%)	0.344
Segment Occlusion (Number (%))				<0.001 *
Supraclinoid Internal Carotid Artery	16 (7.17%)	14 (9.27%)	2 (2.78%)	
Middle Cerebral Artery, M1 segment	162 (72.65%)	122 (80.79%)	40 (55.56%)	
Middle Cerebral Artery, Proximal M2 segment	60 (19.3%)	24 (11.3%)	36 (36.4%)	
Relevant Time Parameters				
Door to CT time (in minutes, mean ± standard deviation)	61.13 ± 144.10	65.72 ± 169.41	52.05 ± 74.02	0.645
Door-to-groin puncture time (in minutes, mean ± standard deviation)	211.50 ± 208.26	212.30 ± 224.78	209.59 ± 166.50	0.959
CT-to-groin puncture time (in minutes, mean ± standard deviation)	174.68 ± 174.67	172.60 ± 180.94	179.50 ± 163.16	0.878
Door to recanalization time (in minutes, mean ± standard deviation)	404.06 ± 419.30	346.49 ± 335.381	542.77 ± 559.01	0.065
CT Perfusion Parameters				
Compensation Index (CI)	2.24 ± 1.39	2.03 ± 1.04	2.67 ± 1.85	*p* = 0.001
Hypoperfusion Intensity Ratio (HIR)	0.38 ± 0.22	0.41 ± 0.21	0.32 ± 0.22	*p* < 0.05
Cerebral Blood Volume (CBV) Index	0.79 ± 0.16	0.78 ± 0.15	0.80 ± 0.16	*p* = 0.42

Statistically significant difference was assessed using unpaired student *t*-test for continuous variables and Chi-square test for categorical variables. Statistically significant results are highlighted with asterisks *.

**Table 2:** Logistic regression model including Compensation Index (CI) in predicting good collateral status as defined by DSA ASITN CS of 3 or greater.

Variables	Univariate Analysis Unadjusted Odds Ratio (OR (95% Confidence Interval))	Multivariate Logistic Regression Analysis			
		Adjusted OR	95% Confidence Interval		*p* Value
			Lower	Upper	
Compensation Index (CI)	1.39 (1.10–1.74)	1.387	1.090	1.766	0.008
Age	0.99 (0.98–1.01)	0.989	0.966	1.013	0.366
Sex	0.80 (0.49–1.30)	0.932	0.493	1.761	0.827
Race	0.99 (0.72–1.40)	0.917	0.579	1.453	0.714
Hypertension	0.74 (0.41–1.27)	0.625	0.289	1.354	0.234
Hyperlipidemia	1.05 (0.65–1.70)	1.136	0.599	2.155	0.696
Diabetes Mellitus	1.16 (0.68–2.00)	1.159	0.555	2.423	0.694
Heart Disease	1.01 (0.63–1.64)	0.914	0.446	1.873	0.806
Atrial Fibrillation	1.17 (0.72–1.91)	1.548	0.740	3.236	0.245
Prior Stroke or Transient Ischemic Attack	1.06 (0.59–1.91)	1.048	0.486	2.258	0.906
Intravenous Tissue Plasminogen Activator Administered (IV tPA)	1.24 (0.76–2.04)	1.298	0.676	2.492	0.433
Admission National Institute of Health (NIH) Stroke Scale	0.96 (0.93–0.99)	0.957	0.912	1.005	0.076
Premorbid Modified Rankin Score (mRS)	1.17 (0.94–1.46)	1.312	0.963	1.787	0.085
Admission Alberta Stroke Program Early CT Score (ASPECTS)	1.25 (1.06–1.45)	1.143	0.937	1.395	0.188

**Table 3:** Logistic regression model including Hypoperfusion Intensity Ratio (HIR) in predicting good collateral status as defined by DSA ASITN CS of 3 or greater.

Variables	Univariate Analysis Unadjusted Odds Ratio (OR (95% Confidence Interval))	Multivariate Logistic Regression Analysis			
		Adjusted OR	95% Confidence Interval		*p* Value
			Lower	Upper	
Hypoperfusion Intensity Ratio (HIR)	3.37 (2.0–5.65)	0.378	0.081	1.757	0.215
Age	0.99 (0.98–1.01)	0.990	0.967	1.013	0.383
Sex	0.80 (0.49–1.30)	0.957	0.504	1.816	0.893
Race	0.99 (0.72–1.40)	0.869	0.553	1.365	0.542
Hypertension	0.74 (0.41–1.27)	0.626	0.293	1.339	0.227
Hyperlipidemia	1.05 (0.65–1.70)	1.140	0.608	2.139	0.683
Diabetes Mellitus	1.16 (0.68–2.00)	1.077	0.523	2.219	0.840
Heart Disease	1.01 (0.63–1.64)	0.885	0.435	1.801	0.736
Atrial Fibrillation	1.17 (0.72–1.91)	1.588	0.773	3.262	0.208
Prior Stroke or Transient Ischemic Attack	1.06 (0.59–1.91)	1.030	0.481	2.205	0.940
Intravenous Tissue Plasminogen Activator Administered (IV tPA)	1.24 (0.76–2.04)	1.163	0.610	2.217	0.646
Admission National Institute of Health (NIH) Stroke Scale	0.96 (0.93–0.99)	0.960	0.914	1.009	0.108
Premorbid Modified Rankin Score (mRS)	1.17 (0.94–1.46)	1.308	0.964	1.775	0.085
Admission Alberta Stroke Program Early CT Score (ASPECTS)	1.25 (1.06–1.45)	1.168	0.959	1.423	0.122

## Data Availability

Data are contained within the article.

## Abbreviations

AIS	Acute ischemic stroke
CI	Compensation Index
CS	Collateral status
CBV	Cerebral blood volume
DWI	Diffusion-weighted imaging
HIR	Hypoperfusion intensity ratio
LVO	Large vessel occlusion
mRS	Modified Rankin score
MT	Mechanical thrombectomy
